# Can spirometry improve the performance of cardiovascular risk model in high-risk Eastern European countries?

**DOI:** 10.3389/fcvm.2023.1228807

**Published:** 2023-08-29

**Authors:** Tatyana Court, Naděžda Čapková, Andrzej Pająk, Abdonas Tamošiūnas, Martin Bobák, Hynek Pikhart

**Affiliations:** ^1^ RECETOX, Faculty of Science, Masaryk University, Brno, Czechia; ^2^ Environmental and Population Health Monitoring Centre, The National Institute of Public Health (NIPH), Prague, Czechia; ^3^ Department of Epidemiology and Population Studies, Faculty of Health Sciences, Institute of Public Health, Jagiellonian University Medical College, Krakow, Poland; ^4^ Laboratory of Population Research, Institute of Cardiology, Lithuanian University of Health Sciences, Kaunas, Lithuania; ^5^ Research Department of Epidemiology and Public Health, University College London, London, United Kingdom

**Keywords:** pulmonary function test, cardiovascular disease, mortality, risk prediction model, cohort studies

## Abstract

**Aims:**

Impaired lung function has been strongly associated with cardiovascular disease (CVD) events. We aimed to assess the additive prognostic value of spirometry indices to the risk estimation of CVD events in Eastern European populations in this study.

**Methods:**

We randomly selected 14,061 individuals with a mean age of 59 ± 7.3 years without a previous history of cardiovascular and pulmonary diseases from population registers in the Czechia, Poland, and Lithuania. Predictive values of standardised *Z*-scores of forced expiratory volume measured in 1 s (FEV1), forced vital capacity (FVC), and FEV1 divided by height cubed (FEV1/ht^3^) were tested. Cox proportional hazards models were used to estimate hazard ratios (HRs) of CVD events of various spirometry indices over the Framingham Risk Score (FRS) model. The model performance was evaluated using Harrell’s C-statistics, likelihood ratio tests, and Bayesian information criterion.

**Results:**

All spirometry indices had a strong linear relation with the incidence of CVD events (HR ranged from 1.10 to 1.12 between indices). The model stratified by FEV1/ht^3^ tertiles had a stronger link with CVD events than FEV1 and FVC. The risk of CVD event for the lowest vs. highest FEV1/ht^3^ tertile among people with low FRS was higher (HR: 2.35; 95% confidence interval: 1.96–2.81) than among those with high FRS. The addition of spirometry indices showed a small but statistically significant improvement of the FRS model.

**Conclusions:**

The addition of spirometry indices might improve the prediction of incident CVD events particularly in the low-risk group. FEV1/ht^3^ is a more sensitive predictor compared to other spirometry indices.

## Introduction

In view of the increasing aging population, cardiovascular disease (CVD) remains the major cause of morbidity, mortality, and economic burden accounting for almost one-third of deaths worldwide (1). Existing prognostic risk stratification models have facilitated the identification and subsequent treatment for people at the high risk of this condition (2–7). The majority of prediction models estimate the individual risk of fatal and non-fatal cardiovascular events based on the presence of risk factors (e.g., age, sex, smoking status, cholesterol, and blood pressure levels) and comorbidities including coronary artery disease, hypertension, and diabetes (3, 5–7). While these models have shown a good discriminative ability in the majority of cases, they are less efficient at the individual level in different populations (2, 8, 9). To improve the prediction value of these models, several new biomarkers have been tested demonstrating different results (10–15). The search for new risk factors that can be easily measured in primary care and can facilitate the management of cardiovascular disease particularly in the population at a high risk for this condition is pivotal.

Previous studies have demonstrated the prognostic value of forced expiratory volume measured in 1 s (FEV1) (16–18) and/or forced vital capacity (FVC) (19, 20) in association with all-cause and cardiovascular mortality in different populations and independent from chronic conditions and smoking status (19, 21). Compared to other common risk factors, lung function has a stronger affinity with the risk of mortality and CVD events seen even in people with a relatively modest level of lung function impairment (22, 23). Most studies tested spirometry indices standardised as the percentage of predicted values (%predicted) or *Z*-scores, and it has also been shown that lung function assessment by FEV1 divided by height cubed (FEV1/ht^3^) demonstrated a strong dose–response relationship with cardiovascular mortality (24).

Studies that investigated the additive prediction of lung function to the CVD risk assessment are limited. The study by Lee at al. found the improved performance of the Framingham risk score (FRS) model combined with FVC and FEV1 to predict all-cause mortality in people with moderate risk of death (25). Another study demonstrated the consistent incremental additive percent predicted values of forced expiratory volume in 1 s (FEV1% predicted) for predicting CVD mortality among other 19 new risk factors added to the Systematic Coronary Risk Evaluation (SCORE) model in the Danish population (14). Models including Framingham risk score and SCORE found to be comparable in predicting CVD events (2, 26, 27); however, the performance of the FRS model has not been thoroughly tested in Eastern Europe. This region remains to experience a high prevalence of CVD, leading to an increased burden of CVD mortality compared to Western Europe (28–30). The major factors contributing to high CVD mortality include health behaviours (e.g., unbalanced diet, higher level of tobacco and alcohol use, and inadequate level of physical activity) (28, 31, 32), pronounced disparities in socioeconomic status (29, 33), and environmental factors (34, 35). In light of these concerns, we aimed to investigate the performance of the FRS model and the additive prediction of spirometry indices including FEV1 and FVC *Z*-score transformed and FEV1/ht^3^ to the risk estimation of fatal and non-fatal CVD events among people from Eastern European countries.

## Materials and methods

### Study design and participants

The prospective Health, Alcohol and Psychosocial factors in Eastern Europe (HAPIEE) cohort study has been designed to investigate the risk factors for high rates of mortality and cardiovascular diseases in Eastern European countries (e.g., Czechia, Poland, and Lithuania) (36). It includes randomly selected people with a mean age of 59 ± 7.3 years from population registers in urban centres in Czechia, Poland, and Lithuania (*N* = 26,746). The centres of data collection were located in seven towns in Czechia and in big cities such as Krakow in Poland and Kaunas in Lithuania. Data on age, sex, health status, medical examination, lifestyle, and socioeconomic and psychosocial factors were collected during 2002–2005. The follow-up survey in the Czechia and Poland and the baseline survey in Lithuania were conducted in 2005–2008 with the use of face-to-face computer-assisted personal interviews combined with clinical examination.

The follow-up time was estimated based on deaths occurring until the end of 2020 in Czechia, until 31 July 2017 in Poland, and until 31 March 2019 in Lithuania. Persons with complete follow-up data were included in the study. Participants were censored on the date of CVD event or the end of the study depending on data availability for each country.

### Ethics approval and consent to participate

All participants provided written informed consent. The study was performed in line with the principles of the Declaration of Helsinki. Approval was granted by the Joint UCL/UCLH Committees on the Ethics of Human Research (Committee Alpha), reference 99/0081; the Ethics Committee of the Kaunas Medical University (reference P1-09/2005); and Ethics Committee at the National Institute of Public Health, Prague (reference 2002-01-08/P1).

### Spirometry and predicted values

Spirometry was performed using a Micro-Medical Microplus spirometer. Participants with acute pulmonary infections and illnesses (e.g., vomiting and nausea), recent surgical procedures, and cardiovascular conditions [e.g., myocardial infarction (MI) and stroke] were excluded from testing (37). Two or more measurements of FEV1 and FVC within 150 ml variation were considered for the study (37). For each participant, the highest values of FEV1 and FVC were selected for further analysis.

Predicted values of FEV1 and FVC were obtained for all participants with age and height as main predictors separately for men and women. *Z*-scores of FEV1 and FVC were calculated using the Global Lung Initiative (GLI) equations (38). In addition, FEV1 was standardised by height and defined as FEV1 divided by height cubed (FEV1/height^3^). All three predicted spirometry indices were also expressed at levels of tertiles of their distribution for further analyses. These spirometry indices were selected based on our previous analyses demonstrating a strong relationship between these measures and all-cause and CVD mortalities in our population (24, 39).

### Outcome

The primary outcomes were fatal and non-fatal CVD events, defined as a composite event of cardiovascular mortality including deaths due to coronary heart disease, heart failure, and stroke, and non-fatal CVD events including myocardial infarction (MI) and stroke. The outcome was defined for people without a previous history of coronary heart disease, myocardial infarction, and stroke. Dates of death were obtained from the national death registers in each country. All registers have shown a complete coverage of deaths (36). Cause-specific mortality was based on underlying causes of death, which were determined according to the selection and application rules of ICD-10 maintained by the World Health Organization (WHO). Participants were followed-up until the incident case of stroke or MI, CVD death, or end of the registration period.

### Covariates

Data on covariates were obtained from questionnaires and medical examination (36). The selection of predictors was based on the original FRS model including age, sex, systolic blood pressure (SBP), hypertension and the use of anti-hypertensive therapy, current smoking, diabetes, total cholesterol (TC), and high-density lipoprotein (HDL) concentrations (3). Smoking status was classified as current in case of currently and regularly smoking at least one cigarette per day. SBP was measured three times, with a 2-min interval between measurements using a digital blood pressure monitor (Omron M5-I). Cholesterol concentrations were measured in fasting venous blood samples with an enzymatic method. This method was calibrated and validated. We also identified the following self-reported comorbidities: stroke, myocardial infarction, ischaemic heart disease, hypertension (defined as measured blood pressure >140/90 mmHg and/or self-reported treated hypertension), diabetes (treated and/or untreated), asthma, and chronic obstructive pulmonary disease (COPD).

### Statistical analyses

All analyses were performed with Stata (Version 17; StataCorp). Descriptive statistics are presented as means with standard deviations (SDs) or frequencies with proportions.

The FRS scores were derived using the STATA module based on validated predictors and coefficients from the FRS Cox proportional hazards regression model (40). Subsequently, FRS scores were categorised into low CVD risk (FRS < 10%), intermediate CVD risk (FRS 10%–19%), and high CVD risk (FRS ≥ 20%). The FRS model was used for people between 30 and 75 years that is within the age range of HAPIEE cohort participants. Spirometry indices were entered into the model as continuous variables and as categorical variables accounting for the level of lung function impairment with highest tertile as a reference. Linear trend was explored by adding polynomials and splines. Based on the original FRS methods, the association of predictors alone and with addition of spirometry with the risk of CVD events was estimated using Cox proportional hazards regression models stratified by country. We used the robust variance estimator to account for possible interactions between groups and multiple comparisons. Proportional hazards assumptions were confirmed by exploring parallelism of log-negative and log-estimated survival curves for each covariate (Supplementary Figures S3–S5). Hazard ratios (HRs) with their corresponding 95% confidence intervals (CIs) were also estimated by crude (included sex and age) and confounder-adjusted models. Using these data, we assessed whether each type of spirometry indices had an independent predictive value and provided additional information on the risk beyond that predicted by the FRS model.

The discriminative ability of each model was assessed with Harrell’s C-statistic applying cross-validation of predictive powers in training and testing sets (41). However, this measure was less sensitive to compare multiple models with varying number of predictors; therefore, we also employed Bayesian information criterion (BIC) and likelihood ratio (LR) for nested models as evaluation criteria for quantifying added value of spirometry indices (42, 43). Calibration was assessed with the Grønnesby and Borgan test (43–45).

We also conducted separate sensitivity analysis by fitting flexible survival models that provide smooth estimates of the baseline cumulative hazards to allow non-proportional effects of age, sex, and spirometry indices (46–49).

## Results

### Descriptive statistics

**Figure 1:**
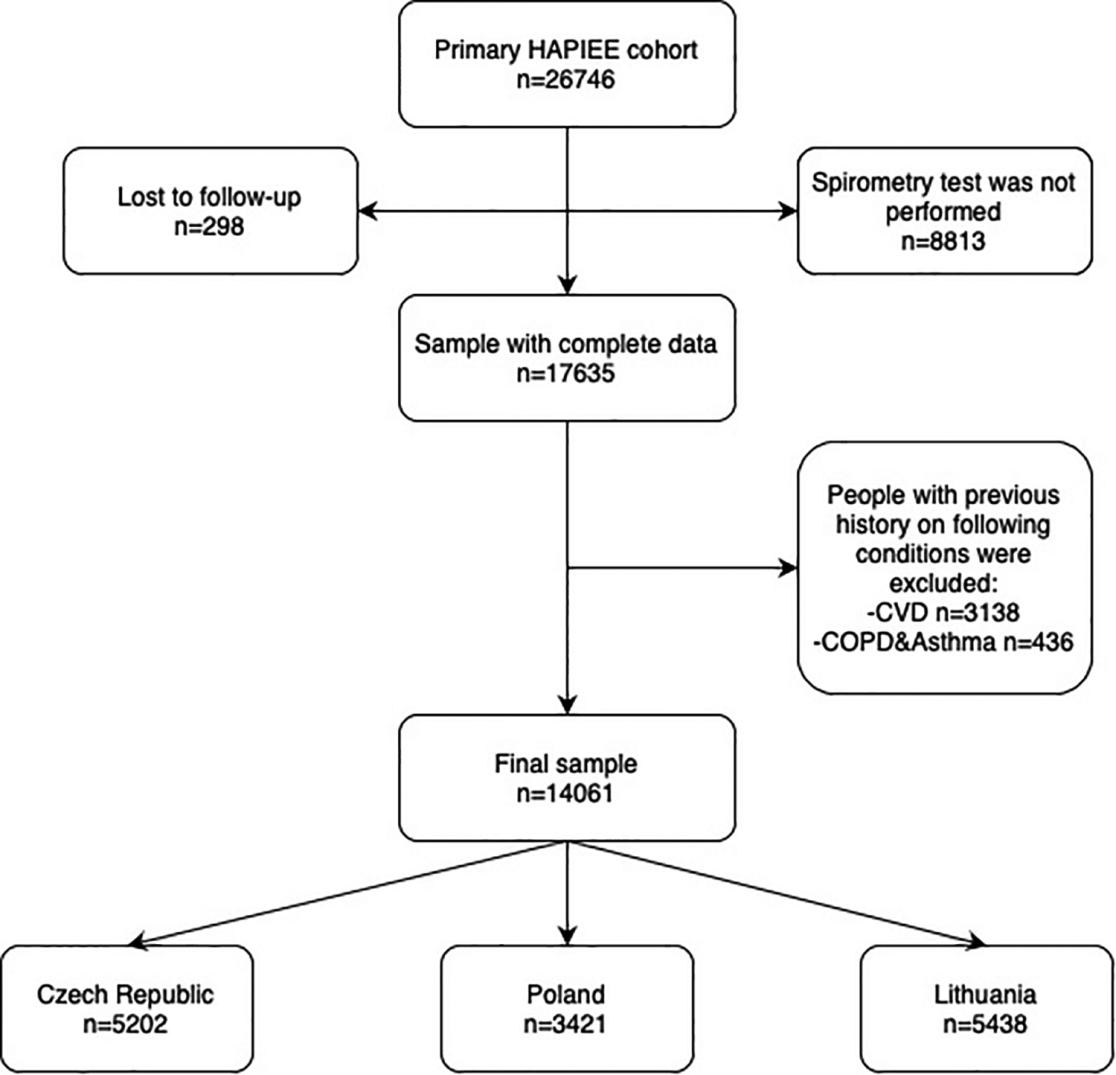
Flowchart of exclusion criteria of the HAPIEE study cohort.

Altogether 26,746 individuals were recruited at baseline, of whom 14,061 met the inclusion criteria (Figure 1). Spirometry tests were performed only on random 50% of respondents in the second year of baseline survey in Poland resulting in a reduced sample size. In addition, non-response in clinical examination among 15%–20% of Czech and Polish participants was another reason for missing data. Data regarding the comparison between countries and spirometry groups are provided in Supplementary Tables 1–2 respectively.

In total, 1,690 CVD events occurred and 987 people died from other causes during the average of 13 years of follow-up (Table 1). CVD events included 463 incident MI, 381 incident strokes, and 846 CVD deaths. Those with CVD events were older (mean age 62 ± 7 years) with a higher prevalence of cardiovascular risk factors and reduced spirometry values (Table 1).

**Table 1:** Descriptive statistics of predictors by cause of death (*n* = 14,061).

	Total	Alive	CVD events	Non-CVD deaths
	(*N* = 14,061)	(81%)	(12%)	(7%)
Age (years), mean (SD)	58.5 (7.4)	57.6 (7.2)	62.4 (6.8)	61.7 (6.9)
Age (years), *n*				
<50	2,383	93.0	4.0	3.0
50–59	5,436	86.1	8.4	5.5
60–69	5,457	73.4	17.1	9.5
≥70	785	61.3	25.8	12.9
Sex, *n*				
Men	6,470	75.4	15.7	8.9
Women	7,591	85.7	8.8	5.5
Country, *n*				
Czechia	5,202	75.1	14.9	10.0
Poland	3,421	90.4	5.2	4.4
Lithuania	5,438	80.6	13.6	5.8
Smoking status, *n*				
Current smoker	3,572	76.6	14.0	9.4
SBP mmHg, mean (SD)	138.2 (20.5)	136.5 (19.8)	147.9 (21.8)	141.8 (21.2)
Total cholesterol mmol/L, mean (SD)	5.8 (1.1)	5.8 (1.1)	5.9 (1.1)	5.7 (1.1)
HDL mmol/L, mean (SD)	1.5 (0.4)	1.5 (0.4)	1.4 (0.4)	1.4 (0.4)
Comorbidities, *n*				
Diabetes	1,163	67.2	21.4	11.4
Hypertension	8,634	76.6	15.4	8.0
Spirometry				
FEV1, mean (SD)	2.8 (0.8)	2.8 (0.7)	2.7 (0.8)	2.6 (0.8)
FVC, mean (SD)	3.5 (0.9)	3.5 (0.9)	3.4 (0.9)	3.4 (1.0)
FEV1 *Z*-score^a^	−0.34 (1.1)	−0.28 (1.1)	−0.56 (1.1)	−0.71 (1.2)
FVC *Z*-score^a^	−0.84 (1.0)	−0.80 (1.0)	−0.97 (1.1)	−1.03 (1.1)
FEV1/ht^3^, mean (SD)	0.56 (0.12)	0.56 (0.12)	0.52 (0.13)	0.51 (0.14)

^a^ The reference values from the GLI with threshold point below lower limit of normal (−1.645).

### Framingham risk score model

The FRS model classified the majority of the study sample as having a low cardiovascular risk (68%), and 18% and 14% of persons comprised the intermediate and high cardiovascular risk groups, respectively (Table 2). Among people with low FRS risk, a substantial proportion were women (78.4%), and the highest number of men were present in the intermediate-risk group (Supplementary Figure S1A). The distribution of CVD risk factors increased in a dose–response manner from low to high CVD risk groups (Table 2). The *Z*-score standardised spirometry indices showed a similar trend with slight decrease in values towards the high-risk group, whereas the highest values of lung function measured by FEV1/ht^3^ was seen in the intermediate-risk group (Table 2). The overall ability of the FRS model to discriminate events from non-events was good and comparable with other populations (C-statistic: 0.714).

**Table 2:** Descriptive statistics of predictors by the Framingham risk score (*n* = 14,061).

	Total sample	Low risk	Intermediate risk	High risk
		<10%	10–20%	>20%
	(*n* = 14,061)	(68%)	(18.4%)	(13.6%)
Age (years), mean (SD)	58.5 (7.4)	57.2 (7.4)	59.6 (6.7)	63.4 (5.9)
Age (years), *n*				
<50	2,383	87.2	10.7	2.1
50–59	5,436	71.1	20.1	8.8
60–69	5,457	58.4	20.0	21.6
≥70	785	52.9	19.7	27.4
Sex, *n*				
Men	6,470	31.8	38.6	29.6
Women	7,591	98.6	1.3	0.1
Country, *n*				
Czechia	5,202	68.5	17.7	13.8
Poland	3,421	68.2	18.8	13.0
Lithuania	5,438	67.2	18.9	13.9
Smoking status, *n*				
Current smoker	3,572	52.6	21.6	25.8
SBP mmHg, mean (SD)	138.2 (20.5)	132.3 (18.2)	144.7 (17.2)	158.9 (19.5)
Total cholesterol mmol/L, mean (SD)	5.8 (1.1)	5.8 (1.1)	5.8 (1.1)	6.0 (1.1)
HDL mmol/L, mean (SD)	1.5 (0.4)	1.5 (0.4)	1.3 (0.4)	1.2 (0.3)
Comorbidities, *n* %				
Diabetes	1,163	46.2	18.5	35.3
Hypertension	8,634	55.3	23.5	21.2
Spirometry				
FEV1, mean (SD)	2.8 (0.8)	2.6 (0.7)	3.2 (0.7)	2.9 (0.6)
FVC, mean (SD)	3.5 (0.9)	3.3 (0.9)	4.0 (0.8)	3.8 (0.7)
FEV1 *Z*-score^a^	−0.34 (1.1)	−0.29 (1.1)	−0.32 (1.1)	−0.57 (1.1)
FVC *Z*-score^a^	−0.84 (1.0)	−0.82 (1.0)	−0.80 (1.0)	−1.00 (1.1)
FEV1/ht^3^, mean (SD)	0.56 (0.12)	0.55 (0.12)	0.59 (0.13)	0.54 (0.12)

^a^ The reference values from the GLI with threshold point below lower limit of normal (−1.645).

### Additive value of spirometry over FRS risk factors

The distribution of the cardiovascular risk categories for the FRS model alone and with addition of spirometry indices (FEV1/ht^3^ tertiles) is outlined in Supplementary Figure S2A. The incidence rates for CVD events per 1,000 person-years progressively increased across all FRS risk groups and from highest to lowest FEV1/ht^3^ tertiles (Supplementary Figure S2A). As shown in Supplementary Figure S2A, the overall incidence rate of CVD events in the moderate FRS risk group increased from 15.9 per 1,000 person-years in people with no/low lung function impairment to 23.4 per 1,000 person-years in people with severe lung function impairment. The difference in rates was more pronounced in the high-risk CVD group, where the incidence of CVD events was higher than predicted even in people with decreased spirometry indices at modest levels (Supplementary Figure S2A).

Trends were similar after adjustment for all components of the Framingham risk score with elevated compared to predicted relative risk of CVD events associated with decreasing lung functions (Supplementary Figure S2B).

### Comparison between spirometry indices

The probabilities of CVD event-free survival according to baseline tertiles of FEV1/height^3^, FEV1, and FVC (*Z*-standardised) spirometry indices are presented in Supplementary Figures S4, S5. All spirometry indices remained independent predictors of CVD events after adjustment for all FRS components, with similar effect estimates of continuous measures (per 1 SD) of FEV1/ht^3^ [hazard ratio (HR): 1.10; 95% CI: 1.05–1.16], FEV1 (HR: 1.12; 95% CI: 1.08‒1.16), and FVC (HR: 1.12; 95% CI: 1.08‒1.18) (Table 3). However, the effect of the continuous variable was driven largely by the lowest tertile of the lung function measure. No significant improvements of the original FRS model in the C-statistic were observed in either model (C-statistic for original FRS model: 0.714 vs. for all spirometry indices: 0.715) (Table 3). However, the LR chi-square test and BIC suggested the superiority of models with the addition of spirometry. All models with continuous spirometry indices had a better fit. Both LR and BIC suggest that the model based on Z-transformed FEV1 measurement had a stronger contribution than other spirometry indices (LR: 1,079.8 and BIC: 29,663.6) (Table 3). In terms of calibration, models with added FEV1-based spirometry measures showed the improved separation between observed and expected risks of CVD events in the moderate- and high-risk groups (Supplementary Table S3 and Figure S6).

**Table 3:** Cox model comparison between different spirometry indices for CVD events.

Predictors	FRS old model	FEV1_ht^3^		FEV1_*Z*		FVC_*Z*	
	HR (95% CI)	HR (95% CI)		HR (95% CI)		HR (95% CI)	
Predictors		Continuous	Categorical	Continuous	Categorical	Continuous	Categorical
Age	1.08 (1.07–1.09)	1.08 (1.07–1.09)	1.08 (1.07–1.09)	1.08 (1.07–1.09)	1.08 (1.07–1.09)	1.08 (1.07–1.09)	1.08 (1.07–1.09)
Women	0.64 (0.58–0.72)	0.61 (0.54–0.68)	0.61 (0.54–0.69)	0.63 (0.57–0.70)	0.63 (0.57–0.70)	0.63 (0.57–0.70)	0.63 (0.57–0.70)
SBP	1.01 (1.01–1.01)	1.01 (1.01–1.01)	1.01 (1.01–1.01)	1.01 (1.01–1.01)	1.01 (1.01–1.01)	1.01 (1.01–1.01)	1.01 (1.01–1.01)
BP treatment	1.25 (1.08–1.44)	1.24 (1.07–1.43)	1.25 (1.08–1.44)	1.24 (1.07–1.43)	1.25 (1.08–1.44)	1.24 (1.07–1.43)	1.25 (1.08–1.44)
Smoking	1.68 (1.51–1.88)	1.63 (1.46–1.82)	1.64 (1.47–1.83)	1.59 (1.42–1.78)	1.61 (1.44–1.80)	1.63 (1.46–1.82)	1.65 (1.48–1.84)
Diabetes	1.51 (1.32–1.74)	1.50 (1.30–1.72)	1.50 (1.30–1.72)	1.48 (1.29–1.70)	1.48 (1.29–1.70)	1.47 (1.28–1.69)	1.48 (1.29–1.70)
HDL	0.81 (0.70–0.92)	0.82 (0.71–0.93)	0.82 (0.71–0.93)	0.82 (0.72–0.94)	0.82 (0.72–0.94)	0.83 (0.73–0.95)	0.82 (0.72–0.94)
Total cholesterol	1.06 (1.02–1.11)	1.07 (1.02–1.11)	1.07 (1.02–1.12)	1.07 (1.02–1.12)	1.07 (1.02–1.12)	1.07 (1.02–1.12)	1.07 (1.02–1.12)
Spirometry per 1 SD		1.10 (1.05–1.16)		1.12 (1.08–1.16)		1.12 (1.08–1.18)	
Highest tertile			1.0		1.0		1.0
Medium tertile			0.99 (0.87–1.12)		1.06 (0.93–1.20)		1.01 (0.90–1.15)
Lowest tertile			1.21 (1.06–1.38)		1.32 (1.17–1.49)		1.23 (1.09–1.39)
Harrell’s C-statistic	0.714	0.714	0.715	0.715	0.715	0.714	0.715
LR	1,053.77	1,068.70	1,067.79	1,079.80	1,074.78	1,077.51	1,069.09
BIC	29,680.12	29,674.74	29,685.20	29,663.64	29,678.21	29,665.92	29,683.90
LR test Old vs. New		14.0***	26.0***	21.0***	23.7 ***	15.3***	14.9***

New model: Old FRS risk score model based on age, sex, systolic blood pressure, hypertension, smoking, diabetes, HDL, and cholesterol + spirometry.

***P ≤ 0.001.

Table 4 shows relative risks of cardiovascular events by FRS groups according to increasing tertiles of spirometry indices. People with poorer lung function had a higher risk of CVD events relative to healthy individuals in all categories of FRS risk. In comparison between spirometry indices, the strongest association with CVD events was seen in the model stratified by FEV1/ht^3^ tertiles. Among persons with low FRS, the risk of CVD events was already elevated in people with intermediate lung function impairment (HR: 1.23; 95% CI: 1.01‒1.50) and doubled for those in the lowest FEV1/ht^3^ tertile (HR: 2.35; 95% CI: 1.96‒2.81).

**Table 4:** Association between degree of lung function impairment (tertiles) and CVD event by FRS groups.

Type of groups	No. of persons	No. of deaths	Person-years of follow-up	Deaths per 1,000 person-years (95% CI)	FRS low risk HR (95% CI)	FRS medium risk HR (95% CI)	FRS high risk HR (95% CI)
FEV1/ht^3^ tertiles							
Highest tertile	5,066	472	46,295	10.2 (9.3–11.2)	1.00	1.00	1.00
Intermediate tertile	4,717	516	43,581	11.8 (10.9–12.9)	1.23 (1.01–1.50)	1.13 (0.89–1.43)	1.33 (1.06–1.67)
Lowest tertile	4,278	702	39,342	17.8 (16.6–19.2)	2.35 (1.96–2.81)	1.57 (1.24–1.98)	1.61 (1.28–2.02)
FEV1_*Z* tertiles							
Highest tertile	4,684	466	42,739	10.9 (9.9–11.9)	1.00	1.00	1.00
Intermediate tertile	4,689	518	43 677	11.9 (10.9–12.9)	1.11 (0.93–1.33)	0.93 (0.72–1.20)	1.37 (1.07–1.76)
Lowest tertile	4,688	706	42,803	16.5 (15.3–17.8)	1.63 (1.37–1.94)	1.31 (1.03–1.65)	1.75 (1.38–2.22)
FVC_*Z* tertiles							
Highest tertile	4,681	517	43,512	11.9 (10.9–12.9)	1.00	1.00	1.00
Intermediate tertile	4,688	519	43,896	11.8 (10.8–12.9)	1.11 (0.93–1.32)	0.87 (0.67–1.12)	1.11 (0.88–1.40)
Lowest tertile	4,692	654	41,811	15.6 (14.5–16.9)	1.53 (1.29–1.82)	1.30 (1.03–1.64)	1.27 (1.02–1.58)

FRS risk score model based on age, sex, systolic blood pressure, hypertension, smoking, diabetes, and HDL cholesterol.

Sensitivity analysis comparing survival models with relaxed proportional hazard assumptions for age, sex, and FEV1/ht^3^ are outlined in Supplementary Tables S4–S6 and Supplementary Figures S7, S8. Despite the significant time-varying effect of age, it did not improve the overall performance of the model. Similar results were observed in models with age interactions. Overall, the majority of women had a low risk of CVD events; therefore, the additive prediction of spirometry indices between sexes did not yield a valid comparison.

## Discussion

In this study of 14,061 persons from Eastern Europeans, we found an independent, strong, and dose-dependent association between impaired lung function and fatal and non-fatal cardiovascular events. The association remained after adjustment and at all levels of traditional cardiovascular risk factors. The most informative spirometry markers for predicting CVD events were FEV1 added as a continuous variable and FEV1/ht^3^ stratified by level of lung function impairment. Adding spirometry indices to conventional Framingham risk model did not change the C-statistic, but it significantly improved the prediction in some risk groups.

In this study, we used the Framingham risk score model, a widely used and robust scoring system for predicting 10-year risk of CVD events worldwide (3, 4). The model was extensively validated showing a wide range of C-statistic values (0.56–0.77) across different populations (26, 50–54). In general, this model tends to overestimate the risk of CVD in both sexes from Western European and American populations (26, 54). In our population, the original FRS model showed a good performance with a C-statistic of 0.71. Despite the declining rates in CVD diseases globally, Eastern Europe is still considered a high-risk region in terms of fatal and non-fatal CVD events with the baseline risk factor pattern similar to those from the derivation population (31, 32). These factors might explain a better risk discrimination of this model in our study. Although models like SCORE and SCORE2 are recommended for use in Europe, their performance in Eastern Europe is similar to the FRS, with C-statistic values ranging from 0.60 to 0.73 (7, 55).

Our results are partially consistent with previous studies investigating the ability of spirometry indices to improve the prediction rates of existing risk models (14, 25). Although different cardiovascular risk prediction models were tested, both studies found improved risk stratification in those with intermediate CVD risk. Compared to the study by Lee et al., the difference in risk rates according to the degree of lung function impairment was observed at all risk levels estimated by the Framingham risk score model in our study (25). The fact that reduced spirometry indices predicted increased risk of CVD events even in relatively healthy individuals with moderate lung function impairment could indicate the additive value of lung function in prediction and prevention of these outcomes in our population. Although the risk distribution was similar, comparability of our results might be affected by the difference in methods where all-cause mortality rather than a composite outcome of fatal and non-fatal CVD events was used for risk stratification. In addition, our cohort was older and more homogeneous in terms of ethnicity and pattern of risk factors.

Moreover, the predicting properties of FEV1 and FVC have been evaluated applying different standardisation methods (14, 25). Previous studies categorised lung function impairment using prediction equations based on reference values from the general population (FEV1% predicted and *Z*-score); however, they have some limitations for the use in the elderly population (38, 56–58). We have previously used both approaches in the same population (24), and they both performed similarly; therefore, only *Z*-transformed values were added to the model. Previous studies reported that FEV1 standardised with height might be a more reliable measure for predicting survival in the elderly population (56, 59–61). We have previously also showed a strong dose–response relationship between lung function (defined as FEV1/height^3^) and cardiovascular mortality in our recent study (39). This might explain the fact that the model stratified by FEV1/ht^3^ tertiles was superior to other spirometry indices (e.g., FEV1 and FVC *Z*-transformed) in predicting the risk of CVD events across FRS risk groups in the current study.

The validity of prognostic performance has been evaluated applying different approaches, and the choice of the best model for survival is not straightforward (41, 43, 62, 63). The accuracy of traditional measures including calibration and discrimination might be affected by censoring distribution. The widely used discrimination measure, Harrell’s C-statistic, should be applied in model assessment with a low censoring rate, and the Gönen and Heller K statistic is a better choice in these settings (43, 64). In order to ensure a valid comparison of our data with findings reported in previous studies, we utilised the C-statistic for assessment of model performance and applied cross-validation techniques to increase the accuracy of this measure (41). Other approaches including likelihood-based measures (e.g., LR test and BIC) showed to be more sensitive approaches to assess whether new biomarkers can improve the overall model fit and performance particularly in the comparison of nested model (15, 42, 43, 65). Therefore, these measures were also implemented for model comparison in our study.

### Limitations of the study

Several limitations deserve to be acknowledged. First, the HAPIEE cohort cannot be seen as being fully representative for Eastern Europe. It only includes several urban centres and does not include data on people from rural areas, and thus it does not cover the whole population of the included countries. Second, the overall response rates were moderately high, and participation in the clinical examination was lower among people from the Czechia and Poland. This may have introduced a selection bias. In addition, data on some risk factors including spirometry were not complete. Both these issues might lead to the underestimation of CVD risk in our study. Third, while the study protocols were identical, and the follow-up time was balanced between countries, the self-reported information in the questionnaire may be a source of reporting bias. Fourth, the composite outcome in our study did not include non-fatal CVD events related to peripheral vascular disease and heart failure, which were part of the original FRS model. This limitation may potentially underestimate our findings.

Fifth, the addition of spirometry indices to the conventional FRS model resulted in small increases in the ability to classify risk, as measured by the C-statistic. On the other hand, the improvement in model performance was detected as measured by the LR test and BIC. Such statistics must always be interpreted in the context of clinically meaningful results.

Finally, the numbers of women in the intermediate and high FRS risk groups were too small for investigations with sufficient statistical power; therefore, the comparison results between sexes were not performed in main analysis. Sensitivity analysis did not show any particular trend in the model comparison between men and women (Supplementary Table S5).

## Conclusions

This is the first study investigating the additive value of impaired lung function to predict fatal and non-fatal CVD events in a high-risk population in Eastern European countries. Spirometry indices predicted CVD outcomes with a clear dose–response fashion, at levels of cardiovascular risk estimated by the FRS. These findings suggest that including spirometry into risk prediction may be useful in high-risk populations.

## Data Availability

The data used to conduct the research are available from the corresponding author but restrictions by the register maintainers apply to the availability of these data. Therefore, the data are not publicly available. However, data are available from the authors upon reasonable request and with permission from the register maintainers. Requests to access the datasets should be directed to hynek.pikhart@recetox.muni.cz.
