# Supplementary material for: Can spirometry improve the performance of cardiovascular risk model in high-risk Eastern European countries?
Source: Front Cardiovasc Med. 2023 Aug 29;10:1228807. doi: 10.3389/fcvm.2023.1228807 (PMC10497938; doi:10.3389/fcvm.2023.1228807)

Supplementary data

Supplementary Table 1. Descriptive statistics of predictors by country (n=14 061)

	**Czech Republic (n=5 202)**	**Poland (n=3 421)**	**Lithuania (n=5 438)**
Age (years), mean (SD)	58.3 (7.1)	58.1 (6.9)	60.9 (7.6)
Age (years), %			
< 50	16.5	16.3	11.9
50-59	37.7	42.5	31.8
60-69	44.1	39.4	43.4
≥ 70	1.7	1.9	13.0
Women, %	54.0	50.7	54.5
Smoking status, %			
Current smoker	25.7	30.3	19.6
SBP mmHg, mean (SD)	139.2 (19.7)	138.2 (20.9)	139.6 (21.6)
Total cholesterol mmol/L, mean (SD)	5.7 (1.0)	5.8 (1.1)	6.0 (1.1)
HDL mmol/L, mean (SD)	1.4 (0.4)	1.4 (0.4)	1.5 (0.4)
**Comorbidities, %**			
Diabetes	11.4	12.1	7.6
Hypertension	65.1	60.5	66.5
Spiromety			
FEV1, mean (SD)	2.7 (0.7)	2.7 (0.7)	2.68(0.8)
FVC, mean (SD)	3.5 (0.9)	3.3 (0.8)	3.5 (0.9)
FEV1%predicted (NHANES)[a], mean (SD)	90.8 (16.4)	91.6 (16.3)	97.1 (16.7)
FEV1 Z-score[b]	-0.56 (1.1)	-0.54 (1.1)	-0.14 (1.1)
FVC Z-score[b]	-0.89 (1.0)	-1.10 (1.0)	-0.62 (1.0)
FEV1/height3, mean (SD)	0.53 (0.13)	0.55 (0.12)	0.56 (0.12)

CVD, cardiovascular disease; SBP, systolic blood pressure; HDL, high-density lipoprotein cholesterol; FEV1, forced expiratory volume in 1 second; FVC, forced vital capacity.

[a]National Health and Nutrition Examination Survey (NHANES) III equations.

[b]The reference values from the Global Lung Initiative (GLI) with threshold point below lower limit of normal (-1.645)

Supplementary Table 2. Descriptive statistics of predictors by FEV1/height³ tertiles (n=14 061)

	**Lowest Tertile (n=4 278)**	**Intermediate tertile (n=4 717)**	**Highest tertile (n=5 066)**
Age (years), mean (SD)	62.2 (6.7)	59.0 (7.1)	56.3 (7.1)
Age (years), %			
< 50	6.2	12.9	24.8
50-59	27.2	38.3	44.6
60-69	57.2	43.3	27.5
≥ 70	9.4	5.6	3.2
Women, %	65.3	58.0	36.7
Country, %			
Czech Republic	41.2	36.9	32.5
Poland	24.5	24.9	26.2
Lithuania	34.4	38.2	41.3
Smoking status, %			
Current smoker	25.0	23.8	24.6
SBP mmHg, mean (SD)	142.2 (21.8)	138.6 (20.5)	136.6 (19.6)
Total cholesterol mmol/L, mean (SD)	5.8 (1.1)	5.9 (1.1)	5.9 (1.1)
HDL mmol/L, mean (SD)	1.4 (0.4)	1.4 (0.4)	1.5 (0.4)
**Comorbidities, %**			
Diabetes	14.7	9.6	6.1
Hypertension	71.4	64.8	57.3
Spiromety			
FEV1/height3, mean (SD)	0.41 (0.09)	0.56 (0.03)	0.68 (0.06)

SBP, systolic blood pressure; HDL, high-density lipoprotein cholesterol; FEV1, forced expiratory volume in 1 second; FVC, forced vital capacity.

Supplementary figure 1

a. FRS risk distribution by sex.

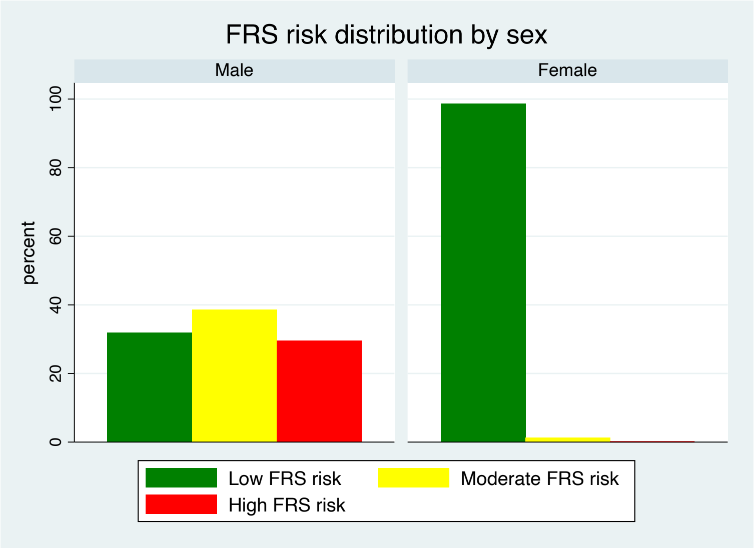

b. FRS risk distribution by FEV1/height^3 tertiles.

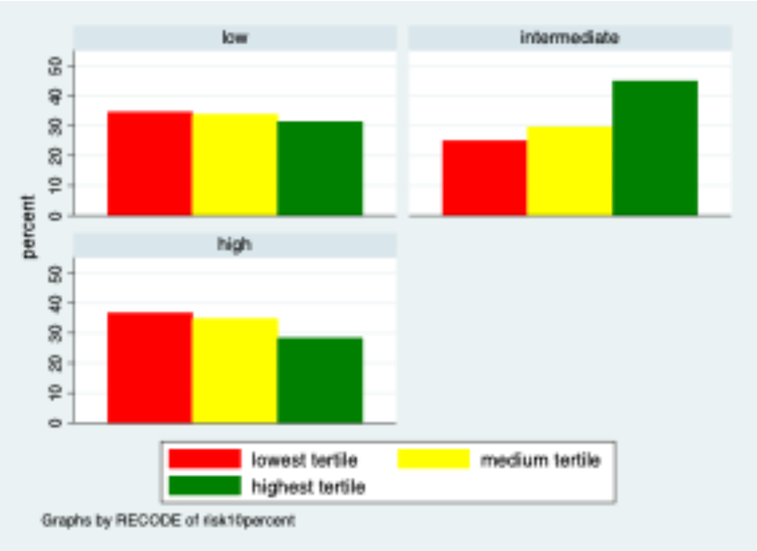

Supplementary figure 2

a. Incidence rates across FRS risk groups by FEV1/height^3 tertiles.

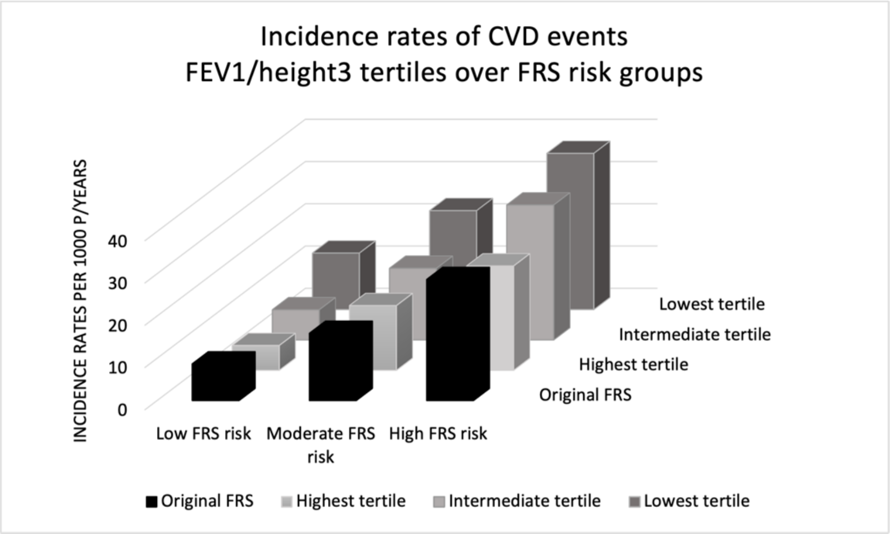

b. Adjusted predictions from crude FRS models vs model with spirometry

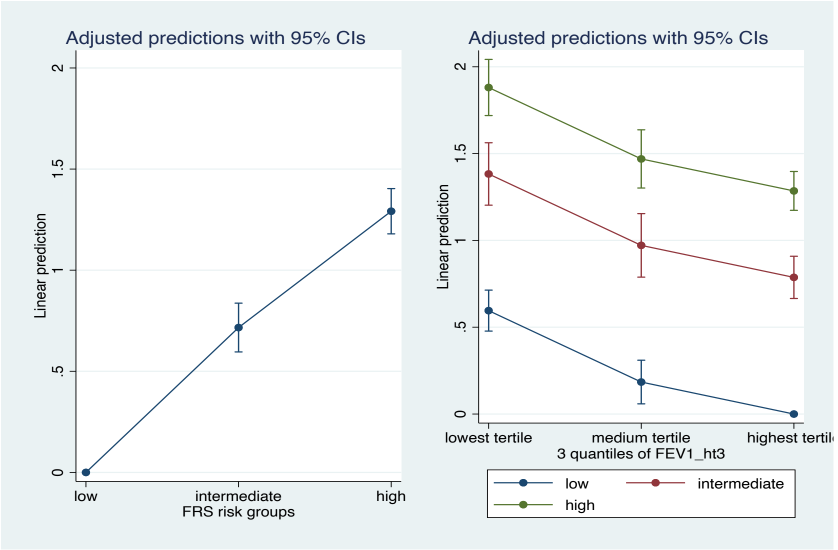

Supplementary Figure 3. Cox proportional-hazard regression model fit.

a. Cox-Snell residuals FRS model

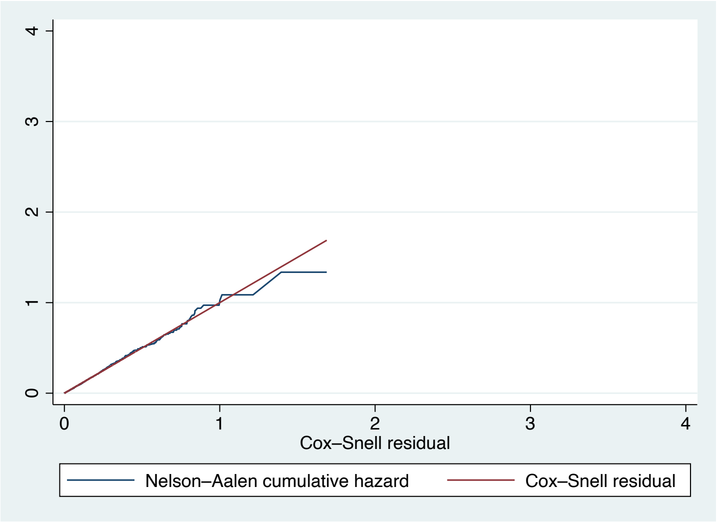

b. Cox-Snell residuals new model (FRS + FEV1/height^3)

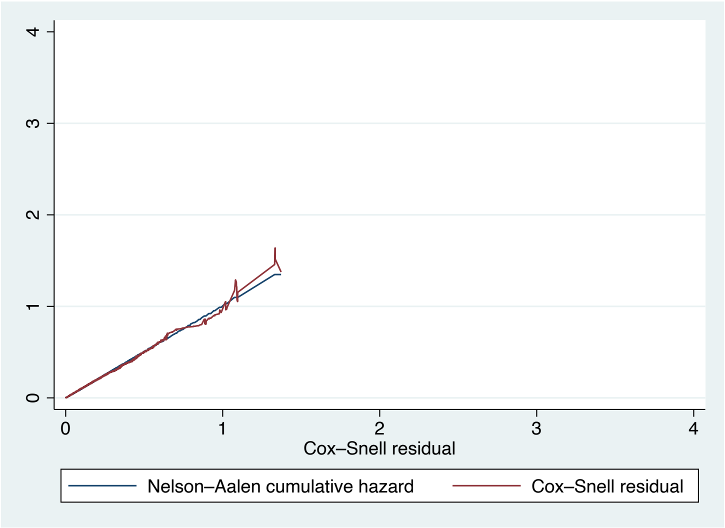

c.PH assumptions (risk score + FEV1_ht3 categorical)

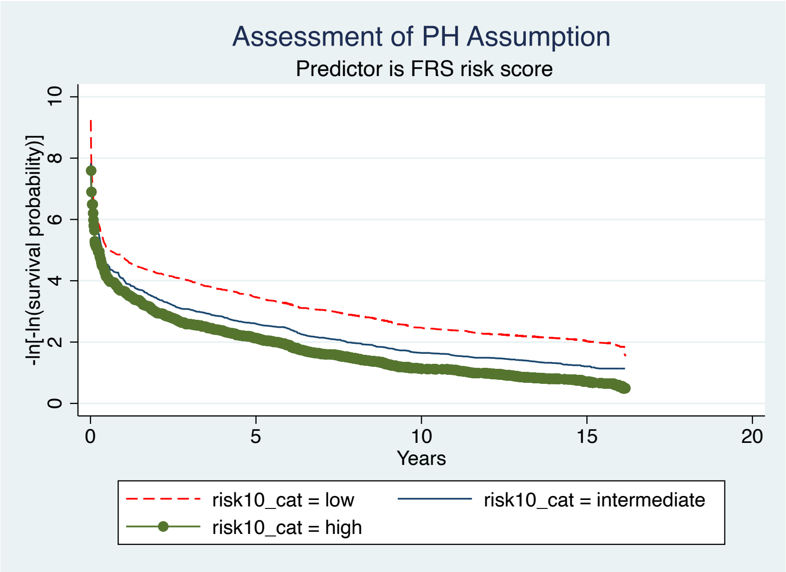

Supplementary Figure 4. KM curves of different spirometry predictors by sex

a. FEV1_ht3 tertiles

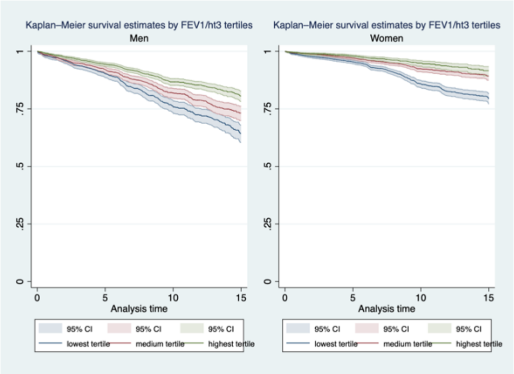

b. FEV1_z tertiles

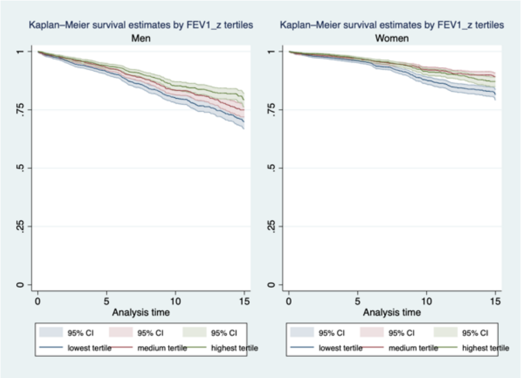

c. FVC_z tertiles

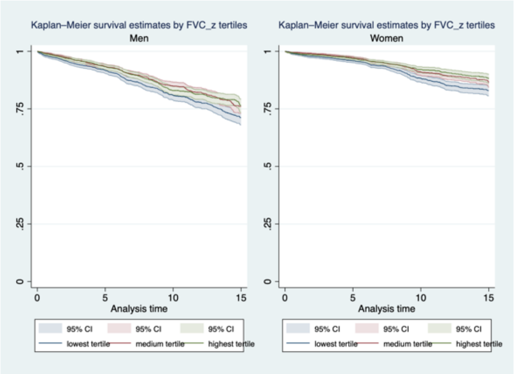

Supplementary Figure 5. KM curves of FRS risk groups by FEV1_ht3_cat

a. Highest tertile

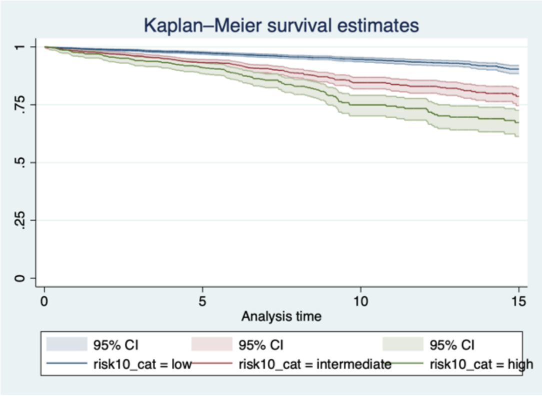

b. Intermdediate tertile

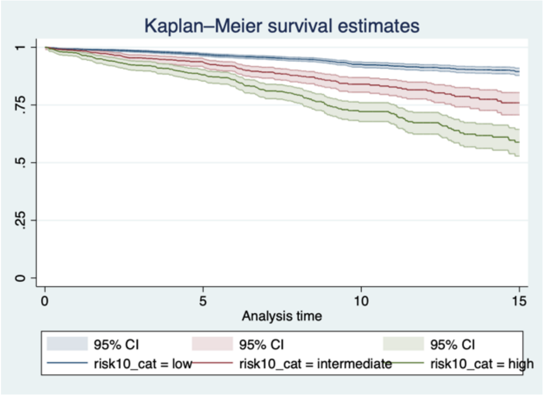

c. Lowest tertile

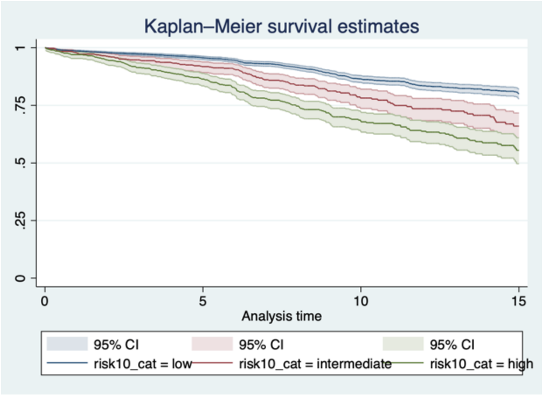

Supplementary Figures 6. Calibration plots based on Grønnesby and Borgan test old model vs new extended model (Cox model)

a.Old FRS model

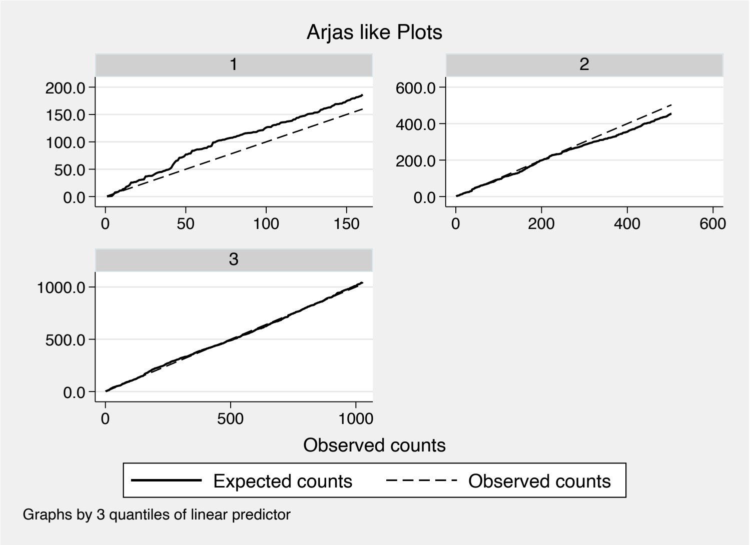

b. New FEV1/height^3

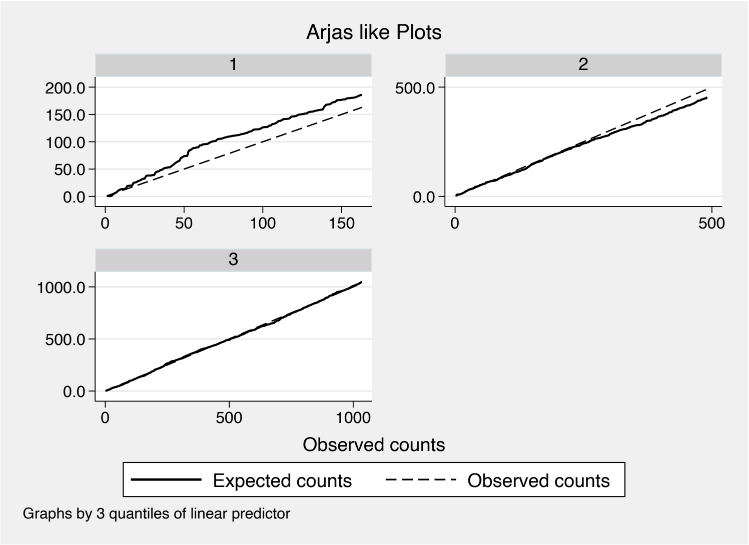

c. ***New FEV1_z***

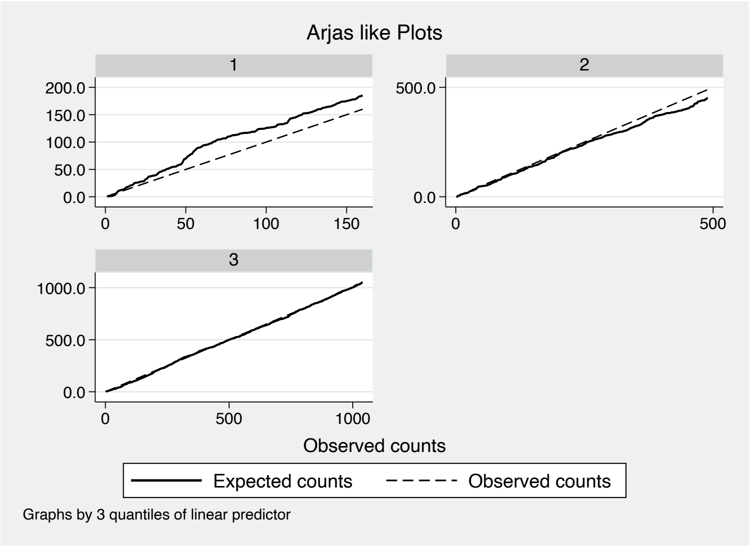

d.New FVC_z

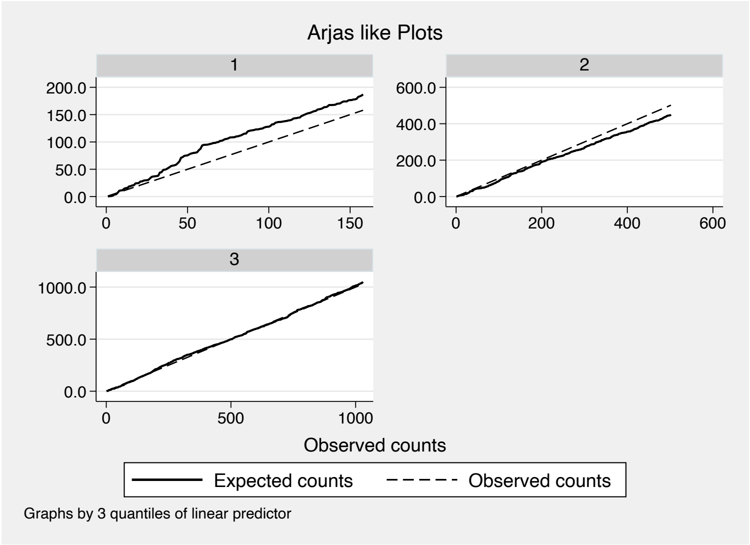

Supplementary Table 3. Model comparison in terms of calibration (based on the Groennesby and Borgan test).

**Risk tertiles**		**I[st] tertile**			**IInd tertile**			**III[d] tertile**	
Models	Observed	Expected	p-value	Observed	Expected	p-value	Observed	Expected	p-value
Crude FRS	160	187	0.05	503	455	0.03	1027	1047	0.53
Extended models:									
FEV1/height³	163	185	0.10	491	453	0.07	1036	1052	0.63
FEV1_Z	160	186	0.06	490	451	0.06	1040	1054	0.67
FVC_Z	158	188	0.03	502	452	0.02	1030	1050	0.53

Supplementary Table 4. Model comparison with age as a time-varying covariate.

Predictors	Crude FRS model HR† (95% CI)	FEV1/height^3 HR‡ (95% CI)	FEV1_Z HR (95% CI)	FVC _Z HR (95% CI)
Age	1.05 (1.04-1.07)	1.05 (1.03-1.06)	1.05 (1.04-1.07)	1.05 (1.04-1.07)
Women	0.64 (0.58-0.72)	0.61 (0.54-0.68)	0.63 (0.57-0.70)	0.63 (0.57-0.70)
SBP	1.01 (1.01-1.01)	1.01 (1.01-1.01)	1.01 (1.01-1.01)	1.01 (1.01-1.01)
BP treatment	1.25 (1.08-1.44)	1.24 (1.07-1.43)	1.24 (1.07-1.42)	1.24 (1.07-1.43)
Smoking	1.69 (1.51-1.88)	1.63 (1.46-1.82)	1.59 (1.42-1.78)	1.63 (1.46-1.82)
Diabetes	1.52 (1.32-1.74)	1.50 (1.31-1.73)	1.49 (1.30-1.71)	1.47 (1.28-1.69)
HDL	0.80 (0.70-0.92)	0.82 (0.71-0.93)	0.82 (0.72-0.94)	0.83 (0.73-0.95)
Total cholesterol	1.06 (1.02-1.11)	1.07 (1.02-1.12)	1.07 (1.02-1.12)	1.06 (1.02-1.11)
Spirometry	-	0.45 (0.30-0.67)	0.89 (0.85-0.93)	0.89 (0.85-0.93)

FRS, Framingham Risk Score model; FEV1, forced expiratory volume in 1 second; FVC, forced vital capacity; HDL, high-density lipoprotein; CI, confidence interval; HR, hazard ratio.

†adjusted for age systolic blood pressure hypertension smoking diabetes HDL cholesterol and stratified by sex.

‡Old model + adjusted for spirometry.

Supplementary Table 5. Model comparison between old (crude model with FRS risk factors) and new (addition of FEV1/height^3) stratified by sex.

Predictors	Old model men HR† (95% CI)	Old model women HR (95% CI)	New model men HR‡ (95% CI)	New model women HR (95% CI)
Age	1.07 (1.06-1.08)	1.10 (1.09-1.12)	1.07 (1.06-1.08)	1.10 (1.08-1.11)
SBP	1.01 (1.01-1.01)	1.01 (1.00-1.02)	1.01 (1.01-1.01)	1.01 (1.01-1.02)
BP treatment	1.12 (0.93-1.35)	1.40 (1.13-1.75)	1.21 (0.92-1.34)	1.39 (1.12-1.73)
Smoking	1.77 (1.56-2.02)	1.50 (1.22-1.85)	1.72 (1.51-1.96)	1.44 (1.17-1.78)
Diabetes	1.45 (1.20-1.74)	1.60 (1.30-1.98)	1.43 (1.19-1.72)	1.59 (1.28-1.96)
HDL	0.82 (0.69-0.98)	0.79 (0.64-0.98)	0.84 (0.70-1.00)	0.80 (0.65-0.99)
Total cholesterol	1.09 (1.03-1.16)	1.01 (0.94-1.08)	1.09 (1.03-1.16)	1.02 (0.95-1.09)
FEV1/height^3	-	-	0.51 (0.31-0.84)	0.34 (0.17-0.68)
Harrell's C-statistics	0.669	0.728	0.669	0.730
Difference in C-indexes	Model for men 0.0015 (0.001-0.002)		Model for women 0.001 (-0.000 0.002)	

FEV1, forced expiratory volume in 1 second; CI, confidence interval; HR, hazard ratio.

†adjusted for age systolic blood pressure hypertension smoking diabetes HDL cholesterol and stratified by sex.

‡Old model + adjusted for FEV1/height^3.

Supplementary Table 6. Model comparison between old (crude model with FRS risk factors) and new (addition of FEV1/height^3) with age interactions.

Predictors	Old model men HR† (95% CI)	Old model women HR (95% CI)	New model men HR‡ (95% CI)	New model women HR (95% CI)
Age	1.15 (1.05-1.25)	1.31 (1.17-1.47)	1.17 (1.05-1.29)	1.37 (1.20-1.55)
SBP	1.03 (1.01-1.06)	1.04 (1.00-1.09)	1.04 (1.01-1.08)	1.05 (1.00-1.09)
*Age*SBP*	1.00 (0.99-1.00)	1.00 (0.99-1.00)	1.00 (0.99-1.00)	0.99 (0.99-1.00)
BP treatment	0.69 (0.14-3.55)	4.01 (0.47-34.0)	0.68 (0.13-3.50)	4.15 (0.50-34.4)
*Age*BP treatment*	1.01 (0.98-1.03)	0.98 (0.95-1.02)	1.01 (0.98-1.03)	0.98 (0.95-1.02)
Smoking	2.37 (0.74-7.57)	1.95 (0.29-12.9)	2.44 (0.76-7.83)	2.34 (0.36-15.1)
*Age*Smoking*	1.00 (0.98-1.01)	1.00 (0.96-1.02)	0.99 (0.98-1.01)	0.99 (0.96-1.02)
Diabetes	8.32 (1.33-52.1)	0.18 (0.01-2.49)	8.77 (1.39-55.2)	0.21 (0.02-2.89)
*Age*Diabetes*	0.97 (0.94-1.00)	1.03 (0.99-1.08)	0.97 (0.94-1.00)	1.03 (0.98-1.07)
HDL	0.48 (0.09-2.46)	0.77 (0.10-5.80)	0.47 (0.09-2.41)	0.77 (0.10-5.68)
*Age*HDL*	1.01 (0.98-1.04)	1.00 (0.97-1.03)	1.01 (0.98-1.04)	1.00 (0.97-1.04)
Total cholesterol	1.41 (0.85-2.36)	2.74 (1.37-5.48)	1.42 (0.85-2.37)	2.66 (1.34-5.28)
*Age*Total cholesterol*	1.00 (0.99-1.00)	0.98 (0.97-1.00)	1.00 (0.99-1.00)	0.99 (0.98-1.00)
FEV1/height^3	-	-	1.24 (0.02-93.4)	4.18 (0.13-24.9)
Age* FEV1/height^3	-	-	0.99 (0.92-1.06)	0.92 (0.83-1.01)

FEV1, forced expiratory volume in 1 second; CI, confidence interval; HR, hazard ratio.

†adjusted for age systolic blood pressure hypertension smoking diabetes HDL cholesterol and corresponding interactions with age stratified by sex

Supplementary Figures 7. Relaxing proportional hazard assumptions for age and spirometry (FEV1/height^3)

a.Time-varying covariate (age)

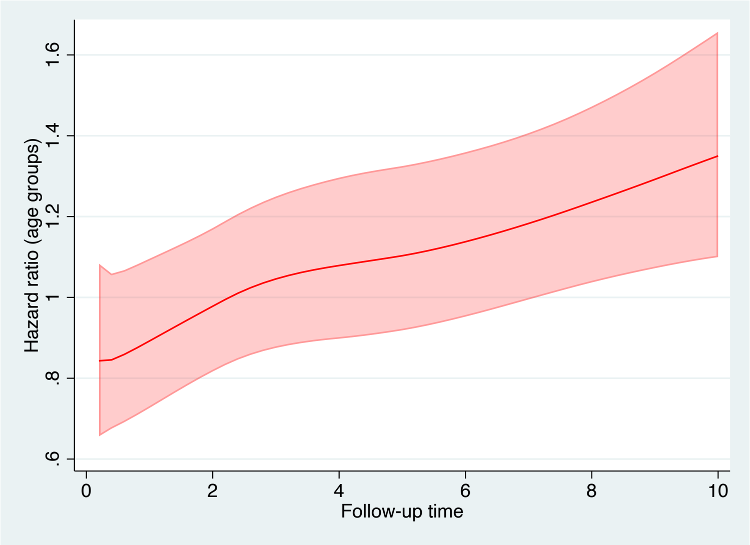

b. Time-varying covariate (*FEV1/height^3*)

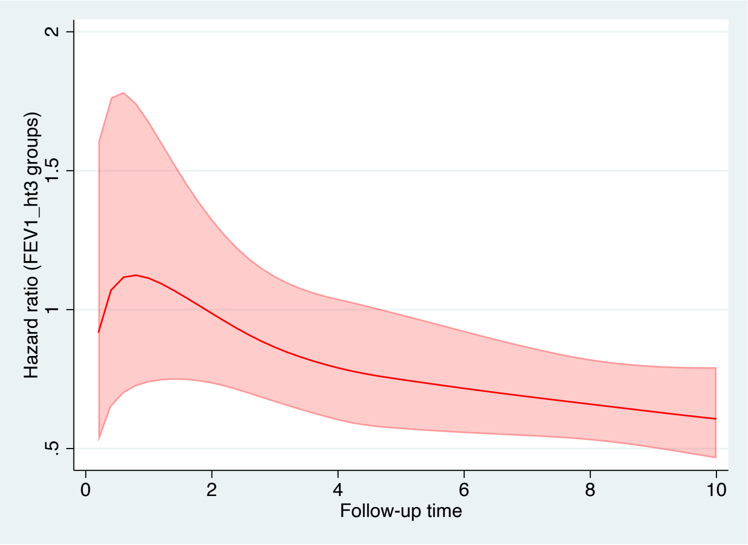

Supplementary Figures 8. Calibration plots old model vs new extended model (parametric stpm2 model)

a.Old continuous

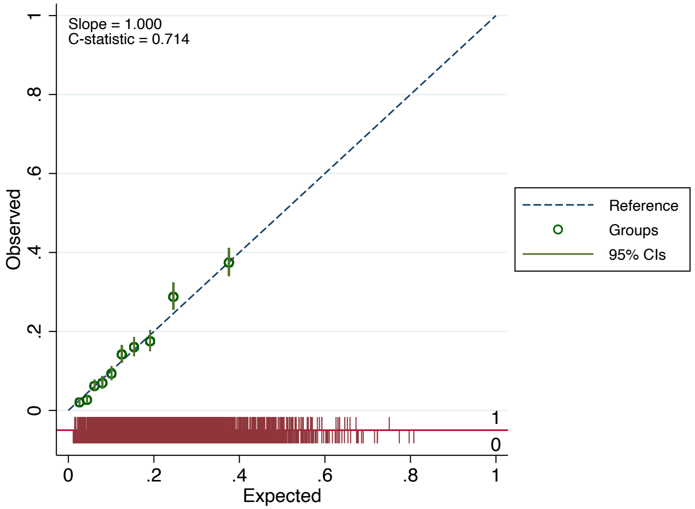

New continuous

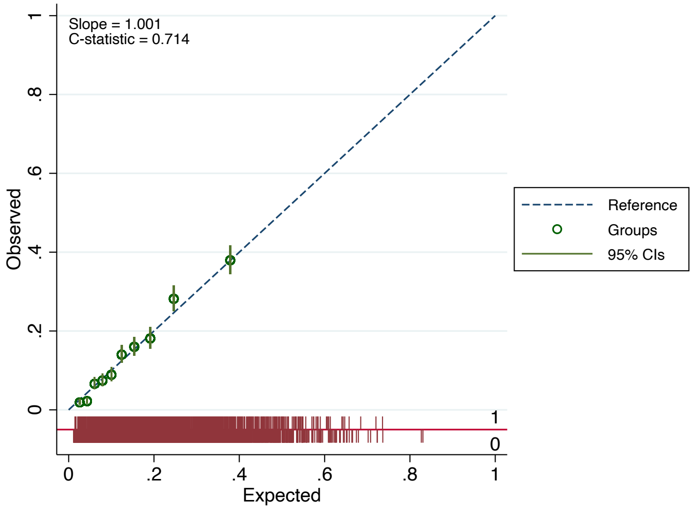

b. ***Old categorical***

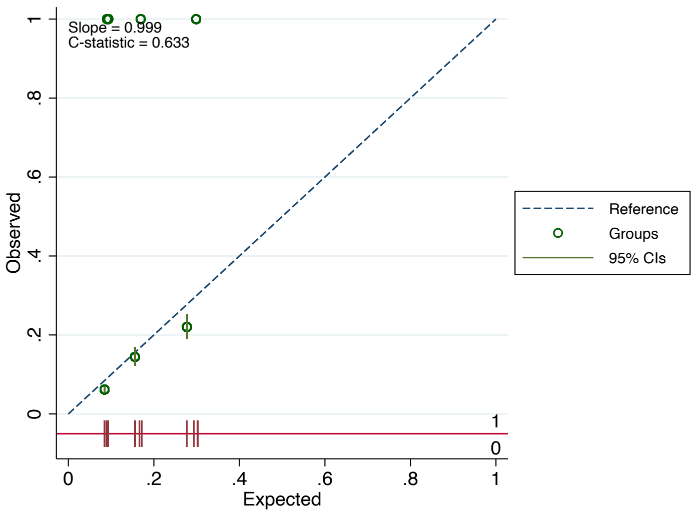

New categorical

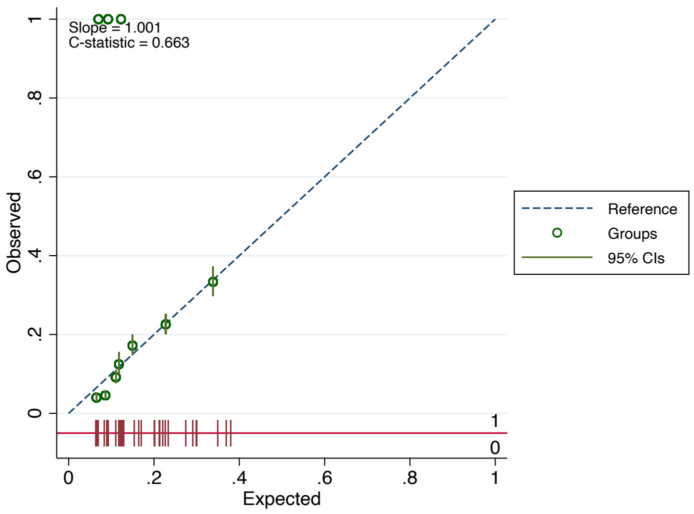

Supplement: Supplementary file 1 [file Table1.pdf]
